# Annual Address of the President before the Medical Society of the County of Erie1Delivered at the meeting of the Medical Society of the County of Erie, held January 10, 1893.

**Published:** 1893-02

**Authors:** William Warren Potter

**Affiliations:** 284 Franklin Street


### ANNUAL ADDRESS OF THE PRESIDENT BEFORE THE MEDICAL SOCIETY OF THE COUNTY OF ERIE.[1]

By WILLIAM WARREN POTTER, M. D.

### INTRODUCTION.

As regularly as the years return come these annual meetings of the Medical Society of the County of Erie, and as 1893 auspiciously begins the cycling of its seasons, it becomes my duty, which is freighted also with pleasure, to welcome you to this meeting and to congratulate you upon its prospects.

The year 1892, just now finished, is, or ought to be, one of pleasant memories to the medical profession of the County of Erie. The remorseless "spirit of the glass and scythe" has touched this Society lightly, and for this we ought to be especially thankful. Only two of its members have gone hence during the year,—one, Dr. John Davidson Hill, in the fulness of his years and in the harvest-time of his professional life, a record of which has been made in due form upon the register of the Society; and the other, Dr. L. C. Carmer,

1. Delivered at the meeting of the Medical Society of the County of Erie, held January 10, 1893.

though not as well known, was a hard-working physician. A suitable tribute to his memory has been prepared and will be spread upon the minutes. Seldom does a year witness a greater degree of prosperity among the people, the general health of the County has been good, and peace and good-will are everywhere prevalent in the ranks of our beloved profession. These, then, are some of the reasons why, when the mantle of office falls upon my successor, I can feel that the year of my service has been marked with as little that could be called unpleasant as generally falls to the lot of the chief officer of this Society.

### STATE MEDICAL EXAMINING AND LICENSING BOARD.

But there are other reasons than those of sentiment that give occasion for rejoicing. It is well to remember that the law creating a separate State Medical Examining and Licensing Board had its origin in this Society. In 1883, special meetings were held and a bill formulated, chiefly through the active interest of Dr. Henry R. Hopkins, that was presented to the Medical Society of the State of New York for its approval. After considerable debate and some modification, the bill was introduced in the legislature through the instrumentality of the legislative committee of the State Society. Here it had a rough and varied career for six or seven years, opposed as it was, I am sorry to say, by a combination of quacks, irregulars, and even some who are orthodox college professors. Finally, however, in 1890, a law was passed, the machinery of the execution of which resides in the Board of Regents of the University of the State of New York.

For the information of those not familiar with the workings of the law, it may not be amiss to recapitulate the essential steps now required of any graduate of 1893 to obtain a license to practise medicine in this State. The conditions required may be grouped under seven heads.

*First.*—The applicant must be a graduate of a college that requires three separate courses of instruction in three separate years.

*Second.*—His diploma must be submitted to the secretary of the Board of Regents, at Albany, with proof of identity, which must also show a competent preliminary education, together with proof of having studied medicine three years, and of having attended three courses of medical instruction in three separate years.

*Third.*—If the preceding conditions are adequately met, the applicant receives a permit from the secretary of the Board of Regents to appear for examination at the next session, held simultaneously in New York, Albany, Syracuse, and Buffalo.

*Fourth.*—The examinations last three days and a half, and are arranged as follows: On Tuesday morning, Anatomy; afternoon, Physiology and Hygiene; Wednesday morning, Chemistry; afternoon, Surgery; Thursday morning, Obstetrics; afternoon, Pathology and Diagnosis; Friday morning, Therapeutics. These examinations are superintended by an examiner, designated by the Regents, but the questions are formulated by the several medical examiners in and for their respective departments.

*Fifth.*—Upon the conclusion of the examinations at any session the Regent's examiner transmits all the papers to the secretary of the Board of Regents, who in turn sends them out to each medical examiner. If all the medical examiners report that the applicant has received a marking of not less than seventy-five per cent. in each and every department, then he is regarded as having passed, and is entitled to a license.

*Sixth.*—Whereupon, a license to practise medicine in the State of New York is issued to the applicant, and he becomes, by virtue thereof, a legal practitioner of medicine and surgery.

*Seventh.*—This license is to be recorded in the County Clerk's office of the County wherein the applicant elects to practise his profession.

Though the law was approved June 5, 1890, to go into operation September 1, 1891, yet under a supplementary exemption act, graduates in medicine who began the study thereof prior to 1889, have escaped its provisions. A further attempt was made in the legislature of 1892 to create exemptions that would have rendered the law practically nugatory for still another year, but this was happily defeated, and I take pride in announcing to you that it is my belief that such defeat was largely accomplished through the efforts of this society.

At a special meeting, held last February, a delegation was appointed to go to Albany, to appear at a public hearing of the students' exemption bill, before the Assembly Committee of Public Health, February 24, 1892. Dr. Edward Clark, one of the delegates, made a forcible address, in which all the objections that the representative of the organized students raised against the bill were answered, and many arguments in favor of maintaining the statute as it is were offered. The speaker of the Assembly himself, Dr. Bush, was present as a listener. It had been previously reported that there was no doubt but that this committee would report the bill to the Assembly, with the recommendation that it pass. I am informed that the committee rose with a tie vote, hence no recommendation was made, and this attempt to modify the law fell just outside the breastworks.

Dr. Ernest Wende, another member of the committee sent by this society, did most serviceable work in acquainting the members of both houses of the legislature, of the direful effects any further exemptions would have upon the progress of medical education in this State. Dr. Bush, the Speaker of the Assembly, I believe, was largely instrumental in preventing any action on this amendment, and I hope this society will take occasion at this meeting to extend its cordial thanks to himself and Dr. Goldberg, which latter also rendered efficient aid in the same direction. Dr. Goldberg is a member of this society, and is also a member of the present legislature; it is to him and through him we must look for the prevention of any further attempts to emasculate the medical practice act of 1890. It now, for the first time, becomes of full force and import to the graduates of medical colleges in this State, as well as to those outside the State who come here to practise. All will be obliged now to submit to the separate State examination before they obtain legal authority to do so.

The points of special importance in this law are: First, that a preliminary education is required before admission to the medical schools. Second, that each medical school in this State must establish a three years' curriculum. Third, a diploma from a legal medical school no longer confers a right to practise, but the holder must submit to a separate examination by the State Board. This releases the medical schools from the dreadful system of rivalry which has heretofore prevailed, and stimulates them to give their students the best equipment possible during three years of collegiate instruction. The effect cannot be otherwise than good upon the profession, and it certainly must benefit the public, but it will take some time to make its full force and effect appreciable in these directions. Ten years' time has been lost through opposition and prejudice; let us endeavor to recover as much of this lost ground as we may by thoroughness and quality. This is the only strong position for the profession to maintain, and it ought to be united in this position.

### HOW SHALL ILLEGAL PRACTITIONERS BE DEALT WITH?

A difficult problem is presented with reference to the practice of medicine by unauthorized or illegal practitioners or persons within our borders, that requires to be met and dealt with by this society very promptly. The Medical Society of the County of New York employs an attorney, who keeps careful watch of its affairs and brings prosecution against these offenders; so, too, with many other medical societies in counties having large cities that offer an inviting field for advertising doctors. The result of all this is that these pretenders flock to Buffalo and infest our community, leaving a disgraceful taint upon the legal profession of medicine. Last year unfortunately Dr. Storck, the efficient Chairman of the Board of Censors, tendered his resignation, which has lain until the present time. Some other members of the board have either formally resigned or declined to act, so that it has been impossible for this society to reach these offenders in an official manner. Lately, it has come to my knowledge that pretenders and illegal practitioners have been carrying on their work in the eastern portion of the city. Through the efforts of Dr. J. F. Krug, a man pretending to practise medicine has been brought to the bar of justice on the verdict of a coroner's jury, which is the first circumstance of the kind within my knowledge or recollection. Lest this opportunity to reach crime should be lost, I authorized Dr. Krug to act for the society in the prosecution of the case, thus assuming the functions of acting chairman of the Board of Censors for the particular purpose in view. I also said to Dr. Krug that he must not involve the society in expense beyond twenty-five dollars without further authority from the society itself. Dr. Krug is prepared to make a report of his acts under this appointment, and I hope upon hearing it the society will feel it a duty and a pleasure to confirm my action. The responsibility was assumed in an emergency about three weeks ago, and, unless it had been taken, the offender probably would have slipped through the meshes of the law, because of the fact that there was no authorized person to follow up the case and present the proper proof to the prosecuting attorney.

In this connection, it comes to my mind, that it would be expedient for this society to employ an attorney to represent it in matters of the kind described. It seems to me that this might be done with a small outlay of money, which would give the society the prestige of an official representative in the courts to look after its interests. If it were once known that we were not only a progressive association of medical men, but that we were aggressive as against all outside illegal practitioners, and that we propose to assert our aggressiveness through a regularly employed attorney, quacks would be slow to enter the boundaries of Erie County, and the dignity of our profession would gain thereby immensely. I offer this suggestion for your thoughtful consideration.

### TRIBUTE TO DR. EDWARD STORCK.

Before ending this portion of my discourse permit me to call the attention of the society to the fact that for twelve years Dr. Edward Storck has served as the indefatigable chairman of the Board of Censors, and his name, during all this period, has been a terror to quacks and impostors who have sought to plant themselves in our midst. In the execution of his duties, it is more than probable that he has incurred the displeasure of many individuals, some of whom may even hold memberships in this society. The task has been a thankless one, and but few men could have been expected to hold the office so long and carry out its functions in so creditable and fearless a manner as has Dr. Storck. It seems to me that the least this society can do is to place upon its minutes some form of appropriate recognition thereof and appreciation therefor.

### THE SECRETARY AND TREASURER OUGHT TO BE PAID.

In order to render the machinery of the society efficient, it seems to me that a small salary should be paid to both the secretary and treasurer for their work, which is concededly of an important character. The discipline of the society is maintained largely, if not absolutely, through the efforts and labor of these two officers. The secretary must not only keep a record of the work done, but he ought to be willing and able to stimulate effective and interesting scientific programmes for our regular meetings. The treasurer must be active in order to secure dues from members, and it ought to be part of his duty to notify new residents in regard to the necessity of becoming members of the society. If he does all this work properly he can easily save more than a salary of fifty dollars, which sum I recommend be paid to both the secretary and the treasurer for their services each year. This can be done by resolution at each annual meeting, and I hope the proposition will meet with the favor of the society, for in order to obtain good work we must pay a reasonable price for it.

### DELEGATES TO THE MEDICAL SOCIETY OF THE STATE OF NEW YORK.

In the selection of delegates to the Medical Society of the State of New York, this body, now entitled to six, can afford to send only such men as manifest an interest in the advancement of the science of medicine, and who will engage to attend the meetings of the State Society as interested official representatives of this society. After three years' service as such, delegates become entitled to permanent membership in a medical body which is concededly the first state medical society of the land.

It seems to me that care should be exercised in the selection of these delegates, so that the society may not be unrepresented nor without a full delegation at any meeting. Under the rules of the State Society, Erie County is now entitled to eight delegates, namely, six representing the Medical Society of the County of Erie, and one each from the medical colleges. If the Buffalo Academy of Medicine is an incorporated society, it also is entitled to representation. There are but fourteen permanent members of the Medical Society of the State of New York who reside in Erie County—too small a number for so large and intelligent a constituency. May I not indulge the hope that Erie County will take pride in increasing this membership as fast as it is permitted to do so under the rules?

### TRANSACTIONS OF THE MEDICAL SOCIETY OF THE STATE OF NEW YORK.

The Medical Society of the County of Erie is expected to subscribe to a number of volumes of Transactions of the State Society equal to five times its representation by delegates. There is an order of the society requiring the Treasurer to attend to this duty every year. The object of this subscription is to supply each delegate, as well as each officer of the society, with a volume, and also such incorporated libraries as exist in the county. The surplus volumes are issued to any member who may take sufficient interest to desire a volume, and who may make an application to the Treasurer for the same. The book is handsomely printed and bound, averaging about 500 pages each year, and it is offered to general purchasers for the sum of $1.50 each. It is doubtful whether more interesting and instructive medical literature is compressed between two book covers, for the same price, than in this annual volume. Attention is invited to the fact, because it is believed to have been overlooked by many who really desire to get these books. This society is an integral part of the State Society, to which it owes allegiance, and is proud to affirm its fealty to such a noble parent. Let us attend its meetings and obtain its Transactions. The one will serve to make us better doctors, the other to enrich our libraries.

### A PROFESSIONAL HOME SUGGESTED.

In a large county like this, one so rapidly increasing in its population and wealth, it seems to me a matter of great regret that the medical societies have no permanent professional home or abiding place. With a number of medical bodies meeting frequently, it seems unfortunate that some must meet in one place and some in another, hence have their books and records scattered in different places. If the several societies would agree to group together in a single place of meeting, which could be rented for a reasonable sum, and where library books and magazines could be found, it would serve to improve the *esprit de corps* of the profession, as well as the social and personal relations of its members to each other. It would be a gratification to many men who have the interest of the profession at heart, to see even such a temporary home leased and established on the basis suggested, which would probably lead to one of permanency in the very near future. This is ot by any means a new thought nor is it propounded as such, but again I invite your attention to the serious consideration of this question, and hope that the initiative of what will lead to the perfection of this plan will be taken by this society. The jurisdiction of this body is limited only by the boundaries of the County of Erie, and such a home would be a pleasant place for the members residing outside of the City of Buffalo to visit whenever they came to town, where they could find a sitting-room for reading, writing, or visiting during unoccupied hours or while waiting for outward-bound trains.

It is quite as much in the interest of the members from the towns that the suggestion is made, as in that of the city contingent, and I hope it will crystallize into a substantial and accomplished fact before many moons have passed.

### THE PAN-AMERICAN MEDICAL CONGRESS OF 1893.

Among the medical meetings appointed for the present year, the chiefest, and that of the most absorbing interest, is the Pan-American Medical Congress, to convene in Washington, September 5, 6, 7, and 8, 1893. At the instance of Dr. Charles A. L. Reed, of Cincinnati, a Committee of Organization, consisting of one from each State and Territory of the United States, with power to perfect the Congress in all its details, was appointed by the American Medical Association, at its Washington meeting, in 1891. This committee held its first meeting in St. Louis, in October of the same year, and laid the foundation for the complete organization of the Congress, by electing Dr. William Pepper, of Philadelphia, President; Dr. Charles A. L. Reed, of Cincinnati, Secretary-General; and Dr. A. M. Owen, of Evansville, Treasurer. Twenty-two sections have been organized, officered by Executive Presidents, English- and Spanish-speaking Secretaries, Advisory Councils, and Honorary Presidents. Vice-Presidents have been chosen from all the constituent countries, and nine Assistant Secretary-Generals have been commissioned. The Congress has been incorporated, a Board of Trustees chosen, and all the necessary steps taken to make the organization legal, strong, and efficient. Congress has authorized the President to invite the constituent countries to send representatives, and it is expected that an appropriation will be made to meet the expenses incident to their entertainment. The preliminary announcement of the Congress, a handsome duodecimo brochure of sixty-four pages, has been issued, a copy of which can be obtained by any physician on application to the Secretary-General.

Every physician of this society is eligible to join the congress, which he can do upon remitting to the treasurer a subscription fee of $10. This will entitle him to all the publications of the congress, including its transactions, which will make several octavo volumes. At the semi-annual meeting of this society, held June 14, 1892, Dr. Reed, the Secretary-General, was present and gave an account of the objects of the congress and the methods of its organization. Many of you, no doubt, will recall his remarks on that occasion.

The following-named gentlemen, residents of Erie County, have been appointed to official positions in connection with the congress, to-wit, Dr. John Cronyn, member of the National Committee of Organization; Dr. A. A. Hubbell, member of the Auxiliary Committee; Dr. William H. Heath, Assistant Secretary-General and Spanish-Speaking Secretary of the Section on General Surgery; Dr. Matthew D. Mann, Honorary President of the Section of Obstetrics; Dr. Henry R. Hopkins, Honorary President of the

Section on Hygiene, Climatology, and Demography; Dr. Charles Cary, Honorary President of the Section on Therapeutics; Dr. Roswell Park, Honorary President of the Section on Orthopedic Surgery; Dr. John Parmenter, Honorary President of the Section on Medical Pedagogics, and Dr. William Warren Potter, Executive President of the Section on Gynecology and Abdominal Surgery. This is a most liberal recognition of the members of this society, and speaks well for the influence which this body exerts in reference to medical affairs of national importance. I hope a considerable number of its members will promptly join the congress by sending their subscription fee to the treasurer, and thus testify their appreciation of its acts as well as their sympathies with its purposes.

### MISCELLANEOUS AND CONCLUSION.

It has been said that interest in the meetings of this society flags when there is no contest or quarrel; on the other hand, it has been said also that interest will flag unless something is done at the meetings besides the discussion of questions of medical policy and politics, and to wrangle over personal and individual interests. Here are two distinct parties, one crying out for an improvement in the scientific professional work to be done by the society at its meetings, and another taking no interest in its affairs unless there is a quarrel on.

There is no doubt but that the functions of this society are in part of a legal nature, where all questions of medical polity relating to the profession in this country may and ought to be discussed. There is no doubt, also, but that above and beyond this there is a large opportunity to hold meetings full of scientific interest and value. Acting upon this belief the committee on programme prepared an excellent one for the June meeting, in which nothing was done, either at the morning or afternoon sessions of over three hours each, but to discuss questions of a scientific nature. At this annual meeting some business must needs be done, but there is ample time for other professional work. Let us then pull together as a body of learned, professional men, and make the society one of strength, equal to the importance which the territory of its jurisdiction holds in the State.

Let each member feel interested in all its affairs, whether of a professional or business nature, and bear equally his or her part, in any capacity in which he or she may be able to serve. But, more than all, let all take a personal interest in its meetings, and come with regularity and a determination to watch its affairs, and to give up the little time which is required therefor during each year, with cheerfulness and good nature.

It may be that it would be wise to hold quarterly meetings,—a proposition which I submit for your consideration. These quarterly meetings could be made of unusual scientific interest, and the men residing outside of the limits of the city could, and ought to, contribute their share to the successful holding of any and all meetings that the society orders.

Into your hands, gentlemen, I commit all these subjects, in the belief that my duty on this occasion has been better performed by making the essential interests of the society a subject of consideration, rather than detain you with a professional or scientific paper. The Committee of Arrangements has prepared an interesting group of these for your consideration, and I shall be most happy to bear my part in making the day one of interest and instruction to the members here assembled.

In conclusion, I beg you to accept my most grateful thanks for the honor you have conferred upon me in calling me to assume the functions of your chief executive officer. It is an honor that I especially appreciate as an expression of confidence on the part of my professional friends at home, and of the esteem that they hold me in. My only regret is that I am not able to give a better account of my stewardship. I pray you to attribute my shortcomings to errors of judgment and not of heart, and to regard me now and evermore as your faithful and obedient servant.

284 Franklin Street.

